# New insights into natural products that target the gut microbiota: Effects on the prevention and treatment of colorectal cancer

**DOI:** 10.3389/fphar.2022.964793

**Published:** 2022-08-15

**Authors:** Lu Lu, Jiahuan Dong, Yujing Liu, Yufan Qian, Guangtao Zhang, Wenjun Zhou, Aiguang Zhao, Guang Ji, Hanchen Xu

**Affiliations:** ^1^ Institute of Digestive Diseases, Longhua Hospital, Shanghai University of Traditional Chinese Medicine, Shanghai, China; ^2^ Department of Oncology, Longhua Hospital, Shanghai University of Traditional Chinese Medicine, Shanghai, China

**Keywords:** colorectal cancer (CRC), gut microbiota, natural products, tumorigenesis, immunotherapy

## Abstract

Colorectal cancer (CRC) is one of the most common malignant carcinomas. CRC is characterized by asymptomatic onset, and most patients are already in the middle and advanced stages of disease when they are diagnosed. Inflammatory bowel disease (IBD) and the inflammatory-cancer transformation of advanced colorectal adenoma are the main causes of CRC. There is an urgent need for effective prevention and intervention strategies for CRC. In recent years, rapid research progress has increased our understanding of gut microbiota. Meanwhile, with the deepening of research on the pathogenesis of colorectal cancer, gut microbiota has been confirmed to play a direct role in the occurrence and treatment of colorectal cancer. Strategies to regulate the gut microbiota have potential value for application in the prevention and treatment of CRC. Regulation of gut microbiota is one of the important ways for natural products to exert pharmacological effects, especially in the treatment of metabolic diseases and tumours. This review summarizes the role of gut microbiota in colorectal tumorigenesis and the mechanism by which natural products reduce tumorigenesis and improve therapeutic response. We point out that the regulation of gut microbiota by natural products may serve as a potential means of treatment and prevention of CRC.

## Introduction

Colorectal cancer (CRC) is one of the most common malignant carcinomas worldwide, and CRC has the third highest incidence and mortality rate according to recent global cancer statistics (Sung et al., 2021). Usually, tumours involving the intestine are diagnosed at advanced metastatic stages of disease. Chronic inflammation is one of the strongest risk factors for CRC (Schmitt and Greten, 2021). Inflammatory bowel disease (IBD), including ulcerative colitis (UC) and Crohn’s disease (CD), contributes to CRC (Keller et al., 2019). CRC associated with colitis can progress from inflammation to dysplasia and ultimately to tumour formation (Xu et al., 2020).

Although great efforts have been made in research on CRC treatment, the prognosis of CRC is still poor, and most patients have poor quality of life (Kishore and Bhadra, 2021). There is a strong public health need for effective prevention and intervention strategies for tumorigenesis. The gut flora includes large numbers of diverse species, and rapid research advances in recent years have increased our understanding of these species (Baumann and Jonzier-Perey, 1988). The number of cells in the gut microbiota is approximately twice the number of somatic cells in the human body. Given that each bacterial species is associated with thousands of genes, the gut microbiota genome is many hundred times larger than the human genome, which is typical estimated to contain 20,000 genes (Almeida et al., 2019). With the continued investigation of the pathogenesis of CRC (Song et al., 2015; Xu et al., 2019), the gut microbiota has recently been reported to play a direct role in the development and treatment of CRC (Sougiannis et al., 2019). Colorectal inflammation facilitates the loss of epithelial barrier integrity and promotes the activation of the inflammation-activating transcription factor NF-κB by the intestinal microbiome and its products; activated NF-κB can then accelerate colorectal tumorigenesis by inducing the production of IL-6, TNF-α, and other cytokines (Greten et al., 2004). Some bacterial genera have also been proven to protect against CRC (Appleyard et al., 2011), and this effect may be mediated through the production of metabolites, induction of immunological tolerance, or an ability to outcompete pathogenic bacteria or fungi (Azab et al., 1988). Regulation of dysbiosis reduces CRC development by restoring intestinal epithelial barrier function and modulating inflammatory immune responses (Tilg et al., 2018; Cheng et al., 2020). In addition, the inflammatory cascade mediated by microorganisms profoundly impacts antitumor effects, including those exerted by chemotherapy and immunotherapy (Sougiannis et al., 2019; Shi et al., 2020; Wang et al., 2021a).

Strategies to modulate the gut microbiome have potential for broad application in the prevention of CRC tumorigenesis and the treatment of CRC (Janney et al., 2020). Natural products have shown promise in the treatment of metabolic diseases and tumours by regulating intestinal flora to regulate host physiology and proinflammatory immune responses, which in turn alleviate disease pathology. Theabrownin is effective in the treatment of metabolic disorders *via* its regulation of the gut microbiome (Huang et al., 2019). Omega-3 polyunsaturated fatty acids (PUFAs) have anti-CRC activity, and the increased abundance of several short-chain fatty acid-producing bacteria might be one of the important mechanisms underlying their efficacy (Watson et al., 2018). In addition, intense research has revealed positive effects of the gut microbiome combined with chemotherapy and immunotherapy. Natural products can reduce the side effects of tumour chemotherapy or increase antitumor effects of treatment (Wang et al., 2021b; Yue et al., 2021). They can not only augment the therapeutic efficacy of immunotherapy but also reverse the resistance of CRC to immune checkpoint inhibitors (ICIs) through combined application (Andrews et al., 2021; Zhang et al., 2021). In this review, we outline the mechanism of action by which the gut microbiota participates in tumorigenesis, providing an overview of how natural products decrease tumorigenesis by regulating the intestinal microbiota. We also summarize potential applications of natural products in combination with chemotherapy or immunotherapy. There is no doubt that this review will serve as a foundation for the development of strategies to inhibit inflammatory-cancer transformation and provide new insights into the clinical treatment of CRC.

### The role of the gut microbiota in colorectal tumorigenesis

A decade of microbiome studies have revealed that the gut microbiome plays an important role in colorectal diseases and colorectal neoplasms (Lynch and Pedersen, 2016). Through the analysis of the faecal microbiota of a longitudinal cohort of 2045 faecal samples from IBD patients and control subjects in four countries (Spain, Belgium, the United Kingdom and Germany), it was clear that patients with IBD have distinct gut microbiota profiles compared to healthy controls (Pascal et al., 2017). The levels of *Bacteroides*, Firmicutes, Clostridia, Ruminococcaceae, *Bifidobacterium*, *Lactobacillus*, and *Faecalibacterium prausnitzii* were decreased in patients with IBD, while those of Gammaproteobacteria, *Fusobacterium* and *Escherichia coli*, especially adherent-invasive *E. coli* (AIEC), were increased in patients with IBD (Man et al., 2011; Knights et al., 2013). Similar to the study conducted in Western countries, an analysis of the prevalence of species in IBD patients in Asia showed similar results. The China cohort study (Ma et al., 2018) and Korea research (Eun et al., 2016) showed patterns of gut dysbiosis in IBD patients. Analysis of the gut microbiota of CRC patients also showed that some bacteria, such as *Streptococcus gallolyticus*, *F. nucleatum*, *Escherichia coli*, *B. fragilis* and *E.* faecalis, have a high prevalence in CRC patients compared to the normal population, whereas the levels of genera such as *Roseburia*, *Clostridium*, *Faecalibacterium* and *Bifidobacterium* are decrease in CRC patients (Feng et al., 2015; Gao et al., 2015; Gagnière et al., 2016; Yu et al., 2017a; Shang and Liu, 2018; Zhang et al., 2019a). Clinical data from different countries show typical intestinal ecological imbalances, which exacerbate the progression of colorectal inflammation and promote colorectal tumorigenesis (Shalapour and Karin, 2020), despite their different genetic characteristics and environmental factors.

A study of AOM-DSS-treated germ-free (GF) mice that were transplanted with faecal microbiota from CRC patients and healthy individuals revealed that the abundance of Gram-negative bacteria, including *Bacteroides*, *Parabacteroides*, *Alistipes*, and *Akkermansia*, was strongly positively correlated with increased tumour burden, while the abundance of members of the Gram-positive Clostridiales, including multiple members of *Clostridium* Group XIVa, was strongly negatively correlated with the formation of tumours (Baxter et al., 2014). Mice treated with azoxymethane show higher intestinal dysplasia after exposure to *P. anaerobius* (Tsoi et al., 2017). However, the role of microbes in the context of intestinal carcinogenesis is complex and diverse. We discovered that GF mice developed significantly more and larger tumours than specific pathogen-free (SPF) mice after AOM and DSS treatment. Recolonization of GF mice with commensal bacteria or administration of lipopolysaccharide (LPS) reduced tumorigenesis (Zhan et al., 2013). Thus, although the intestinal microbiome is capable of driving chronic inflammation and tumorigenesis, commensal bacteria also play important roles in limiting chemically induced injury and tumour development.

Inflammation-related signalling pathways, such as the NF-κB pathway (Wu et al., 2018), Toll-like receptor (TLR) pathway (Li et al., 2014), and Wnt/β-catenin pathway (Schatoff et al., 2017), are closely related to the occurrence and development of tumours. Microbes and their products can activate the major inflammation-activated transcription factor NF-κB; then, by inducing the production of IL-6, TNF, and other cytokines, NF-κB can accelerate the development of colon (Greten et al., 2004; De Santis et al., 2015) and pancreatic (Zhang et al., 2013) cancers. The tumour-promoting effects of bacteria can be enhanced by LPS, which is a component of the outer cell wall of Gram-negative bacteria that protects bacteria against antibiotics or immune cells. The binding of LPS and lipopolysaccharide-binding protein (LBP) can increase its affinity for CD14 receptors. Then, the LPS/LBP/CD14 complex binds to myeloid differentiation factor 2 (MD-2), which is recognized by TLR4 (Potrykus et al., 2021). Activation of this receptor leads to the release of mediators, including MyD88, which stimulates NF-κB to produce proinflammatory cytokines, including TNF-α and IL-1β (Gnauck et al., 2016). Another study also showed that LPS promotes CRC progression by activating TLR4-MyD88-NF-κB signalling in response to *Fusobacterium nucleatum* (Fn) (Zhu et al., 2016), which has been reported to be positively associated with CRC carcinogenesis (Gethings-Behncke et al., 2020; Villar-Ortega et al., 2022); this signalling pathway can activate the Wnt/β-catenin pathway through upregulation of cyclin-dependent kinase 5 (Cdk5), thus promoting the proliferation and migration of cells. The *in vitro* results showed that a cocktail of *Lactobacillus spp*. may exert an antitumorigenic effect by downregulating the expression of the genes encoding β-catenin and CTNNB1 and increasing the expression of genes that control the degradation of the β-catenin complex (Ghanavati et al., 2020). In addition, butyrate inhibits inflammation and carcinogenesis by reducing NF-κB and Wnt signalling (Inan et al., 2000; Uchiyama et al., 2016). The inflammatory response mediated by the microbiota can contribute to the inflammatory response through different signalling pathways, thereby regulating inflammation-related cancer progression (Shalapour and Karin, 2020). LPS is one of the important links in the inflammatory response to microorganisms. On the other hand, microbiota and microbial metabolites can reduce tumorigenesis by regulating inflammatory signalling pathways.

The crucial parameters of enteric infections are colonization resistance, microbiome community structure and niche occupation, and these parameters can be modulated by mucus (Sorbara and Pamer, 2019). To colonize the intestinal epithelium, pathogens have to pass through the mucus that is secreted by goblet cells in the proximal colon and distal colon; thus, mucus provides a physical, chemical and biological line of defence for the host (Sauvaitre et al., 2021). Impaired mucosal barrier function is accompanied by decreased acidic mucin expression, decreased mucus layer thickness, and decreased antimicrobial peptide levels, which are associated with gut dysbiosis (Liang et al., 2020). *Bacteroides* and *Akkermansia* are the two genera whose presence is most strongly correlated with higher rates of tumorigenesis. Both are known to degrade mucin, and the expression of several genes associated with mucin degradation is positively correlated with intestinal inflammation (Bloom et al., 2011; Ganesh et al., 2013; Ng et al., 2013) and tumour incidence (Baxter et al., 2014). *Clostridium perfringens* can exhibit proteolytic and mucinase activity and lead to a thinner mucus layer, which plays a positive role in the pathogenesis of colon inflammatory disease (Machiels et al., 2017). Cathelin-related antimicrobial peptide (CRAMP) (Kurosaka et al., 2005), which cannot be detected after 21 days in infants, is expressed in small intestinal epithelial cells during the neonatal period and significantly protects neonates from the enteric pathogen *Listeria* (Ménard et al., 2008). Additionally, tight junctions (TJs) are critical for transepithelial permeability, and they restrict passage of pathogens, microbes or toxins into the host (Groschwitz and Hogan, 2009). Complex crosstalk between the gut barrier and intestinal microbiota regulates not only host homeostasis but also disease development.

Moreover, tumour and microenvironment cells respond to signals from the microbiota. The intestinal microbiome can regulate heterogeneous cell populations, such as endothelial, stromal, and immune cells, leading to the secretion of soluble signals (cytokines, chemokines, or growth factors) and generating a favourable microenvironment to support tumour growth and progression (Lu et al., 2021). An experiment in a APC Min/+ mouse model showed that *F. nucleatum* exacerbates tumorigenesis by recruiting tumour-infiltrating myeloid cells (granulocytes, macrophages, dendritic cells (DCs), and MDSCs), and these mice share a proinflammatory signature with *Fusobacterium*-associated human CRC (Kostic et al., 2013; Hanus et al., 2021). *F. nucleatum* can promote tumorigenesis by decreasing CD3^+^ T cell numbers (Mima et al., 2015). APCMin/+ mice exposed to colibactin-producing *E. coli* exhibit more polyps and decreased CD3^+^ CD8^+^ T cell number than noninfected animals or animals infected with *E. coli* strains that lack pks (Lopès et al., 2020). Enterotoxic *Bacteroides fragilis* (ETBF) indirectly induces the ectopic production of chemokines and growth factors by colonic epithelial cells (CECs) through interaction with IL‐17 receptors. ETBF also induces submucosal IL‐17 expression. IL‐17 and transformed CECs jointly promote tumour development by suppressing immune effector cells and activating the STAT3 signalling pathway, together with MMP‐9 and VEGF (Thiele Orberg et al., 2017). Dysbiosis stimulates macrophages to increase the phosphorylation of c‐Jun in CRC cells and accelerate CRC cell proliferation (Li et al., 2012). These data suggest that bacteria promote a tumour microenvironment (TME) that favours neoplasia progression. Although the mechanism by which the gut microbiota contributes to the TME has not been elucidated, many reports that suggest that the gut microbiota and its metabolites affect antitumor immune responses. A healthy gut microbiome can stabilize T cells that recognize a wide variety of antigens and are activated to differentiate into cytotoxic CD8^+^ T cells, ultimately infiltrating the tumour and attacking tumour cells (Iida et al., 2013). It has been reported that 11 strains present in the intestinal microbiota can increase CD8^+^ T cell numbers, enhance the antitumour immune response mediated by CD8^+^ T cells and inhibit tumour progression. The effect is the same as, or even better than, ICIs (Tanoue et al., 2019). Stimulation of NK cells with *Lactobacillus plantarum* (Lp) enhances IL‐22 production, which decreases damage to the intestinal epithelial barrier (Suzuki, 2013) and delays tumour formation (Niu et al., 2021). Butyrate enhances Treg function in a murine model (Arpaia et al., 2013; Smith et al., 2013).

**FIGURE 1:**
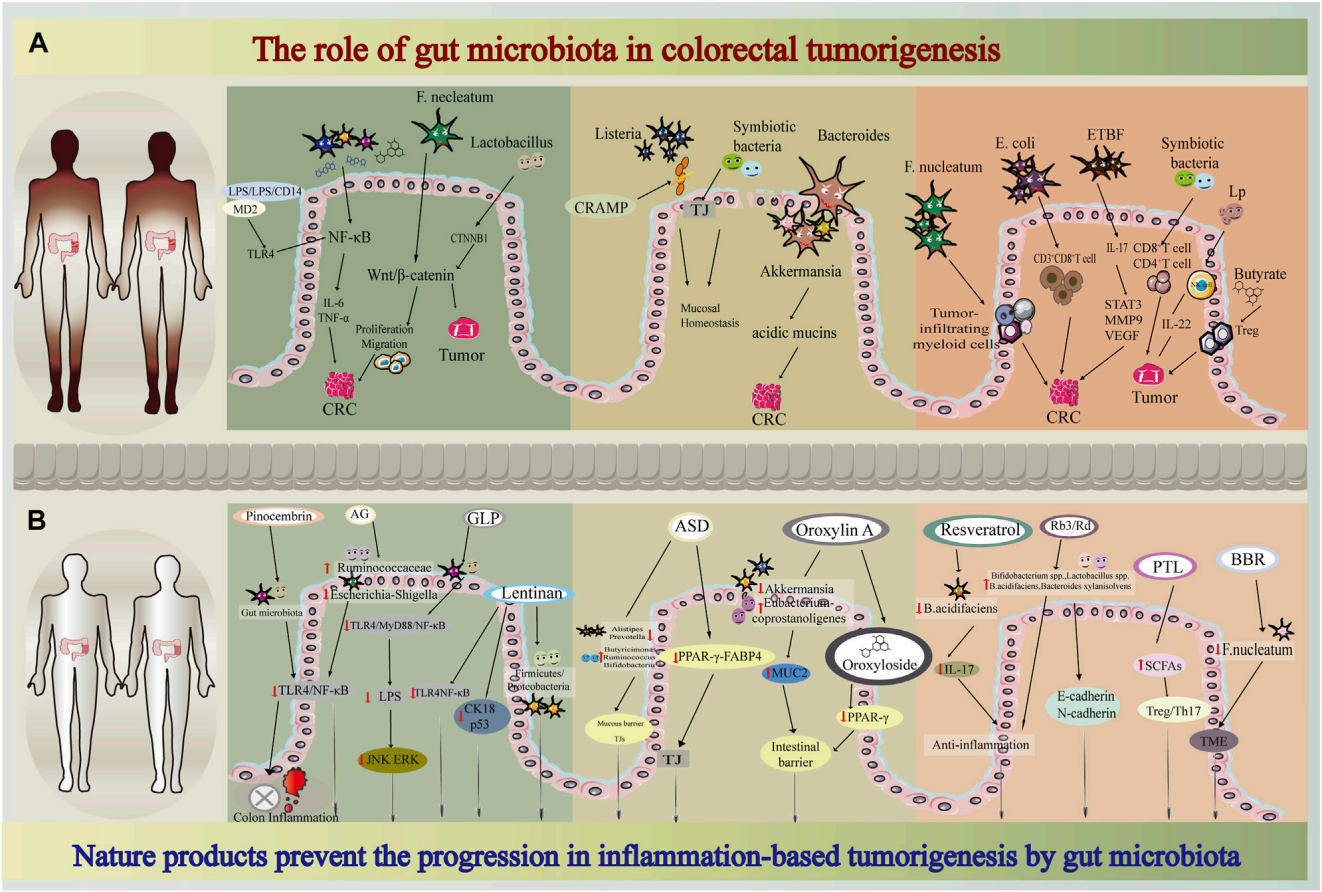
Flow diagram of mechanistic study through nature products, gut microbiota, colorectal tumorigenesis **(A)** The role of gut microbiota in colorectal tumorigenesis **(B)** Nature products prevent the progression in inflammation-based tumorigenesis by gut microbiota.

These studies have shown that the gut microbiota plays an important role in maintaining intestinal homeostasis as well as in the occurrence and development of colorectal inflammatory diseases and tumorigenesis (Figure 1A). Therefore, effective interventions that modulate gut microbes are a powerful strategy to inhibit CRC transformation. Specifically, increasing the commensal flora, reducing the growth of pathogenic bacteria, restoring ecological disturbance, maintaining the intestinal epithelial barrier, and regulating the state of the immune microenvironment decrease colorectal inflammation and tumorigenesis. Natural compounds are strong candidates for achieving these goals.

### Natural products prevent the transformation of colorectal inflammation into colorectal cancer by the gut microbiota.

Natural products play an important role in the regulation of immunity and the treatment of disease. A study has shown that treatment with 300 mg/kg resveratrol alleviates gut dysbiosis by increasing the *Firmicutes/Bacteroidetes* ratio, inhibiting the growth of *Enterococcus faecalis*, and increasing the prevalence of the probiotics *Lactobacillus* and *Bifidobacterium* in the gut (Etxeberria et al., 2015). Resveratrol (0.025%) effectively decreases the abundance of the genera *Akkermansia*, *Dorea*, *Sutterella* and *Bilophila* in mice, increases the proportion of *Bifidobacterium* and increases alpha diversity. Resveratrol administration protects colonic tissue from structural deterioration and dysplasia and reduces the expression of several proinflammatory cytokines. Furthermore, treatment with 1.56 μg/ml (6.8 μm) resveratrol markedly inhibits the biofilm formation of Fn under anaerobic conditions (He et al., 2016). Feeding mice 0.5% curcumin for 14 weeks can significantly increase bacterial richness, prevent age-related decreases in alpha diversity, increase the relative abundance of *Lactobacillales*, and decrease the relative abundance of members of the Coriobacterales order (McFadden et al., 2015), which can exert strong anti-inflammatory, antioxidative and antiproliferative effects. Quercetin supplementation decreases the relative abundance of the potentially pathogenic microbe *E. coli* (Zhang et al., 2017a). Both chlorogenic acid (Zhang et al., 2017a) and salvianolic acid A (Wang et al., 2018a) significantly isolate the microbiota and suppress the progression of colitis and CRC. These natural product-mediated alterations in the gut microbiome provide evidence for a protective role of natural products in inflammatory-cancer transformation. Natural products may regulate the *Firmicutes/Bacteroidetes* ratio, increase faecal counts of potentially beneficial microbes and decrease the relative abundance of potentially pathogenic microbes. There is a dose-dependent trend in the regulation of intestinal flora by natural products, but more dose-ranging studies are needed to determine the optimal dose of natural products.

As we described above, the gut microbiota may regulate the transformation of inflammation into cancer through different inflammatory pathways. Pinocembrin, a plant-derived flavonoid, alleviates UC in mice by regulating the gut microbiota and suppressing the TLR4/MD2/NF-κB pathway (Yue et al., 2020). *Ganoderma lucidum* (GLP) was proven to be an effective natural product that protects against AOM/DSS-induced inflammation. GLP alleviates endotoxaemia by inhibiting TLR4/MyD88/NF-κB signalling, ultimately suppressing inflammatory marker expression and MAPK (JNK and ERK) activation (Guo et al., 2021). A mouse model showed that treatment with astragalin (AG) ameliorates metabolic endotoxaemia, improves intestinal mucosal barrier function, and increases the abundance of potentially beneficial bacteria (such as Ruminococcaceae) and decreases the abundance of potentially harmful bacteria (such as *Escherichia* and *Shigella*). Further experiments showed that AG inhibits the relative mRNA expression levels of TLR4 and inhibits NF-κB pathway activation (Peng et al., 2020a). Treatment with α-ketoglutarate, an important intermediary in the NF-κB-mediated inflammatory pathway, tends to minimize the proportion of opportunistic pathogens (*Escherichia* and *Enterococcus*) while increasing the proportion of *Akkermansia*, *Butyricicoccus*, *Clostridium*, and *Ruminococcus* and protecting against inflammation-related CRC (Li et al., 2019). Another study showed the therapeutic potential of lentinan in mouse models of IBD and CAC; lentinan exerts its beneficial effect in mice with IBD and CAC possibly by inhibiting TLR4/NF-κB signalling and the expression of colon cancer markers. 16S rRNA gene sequencing confirmed that lentinan treatment restores the Firmicutes/Proteobacteria ratio to nearly normal levels (Liu et al., 2019). Upregulated expression of TLR4 is a common feature in tissues from IBD and CRC patients (Burgueño et al., 2021). In human tissue microarrays, TLR4 expression increases specifically in CECs as tissues progress from normal to neoplastic stages (Santaolalla et al., 2013; Sussman et al., 2014), and epithelial TLR4 deficiency protects mice from CAC development (Fukata et al., 2009). NF-κB is a key regulator of inflammation, innate immunity, and tissue integrity (Banoth et al., 2015). Natural products can regulate inflammatory pathways and reduce the serum levels of LPS and proinflammatory cytokines (COX-2, MCP-1, TNF-α, IL-6, IL-1β, and IFN-γ), which contribute to suppressing tumorigenesis. On the other hand, natural products can also induce antitumor immunity by regulating the transformation of inflammation, which will be discussed later.

Increasing numbers of studies reveal that traditional herbal extracts have a positive relationship with decreased mortality due to a variety of chronic diseases, such as cardiovascular disease, cancer and diabetes (Wang et al., 2020a; Bu et al., 2020). However, the physiological effects are in marked contrast to their poor bioavailability (Zhao et al., 2020; Zhi et al., 2020). Products with poor bioavailability may serve as potential substrates for the gut microbiota. These products exert therapeutic effects by modulating the composition of the gut microbiome and affecting the gut barrier (Feng et al., 2019). Akebia saponin D (ASD) significantly modifies the gut microbiome; it reduces the proportions of *Alistipes* and *Prevotella* and enhances the proportions of *Butyricimonas*, *Ruminococcus*, and *Bifidobacterium*. RNA sequencing (RNA-seq) revealed that ASD reduces lipid-induced TJ damage in intestinal epithelial cells *via* downregulation of the PPAR-γ-FABP4 pathway *in vitro* and that the PPAR-γ inhibitor (T0070907) partially blocks the effects of ASD (Yang et al., 2021). Experiments in which colitis was induced by DSS administration demonstrated that oroxylin A, a natural flavonoid, reduces susceptibility to colitis and prevents carcinogenesis in colon (Jung et al., 2012). Oroxylin A upregulates the mRNA level of Muc2 *in vivo*. Analysis of the gut microbial composition showed that treatment with oroxylin A decreases the abundance of *Akkermansia*, which has been confirmed to contribute to the breakdown of the mucus layer (Zhai et al., 2019; Wang et al., 2021c). Additionally, the abundance of *Eubacterium coprostanoligenes* is increased by oroxylin A, contributing to the protection of the colon against colitis and carcinogenesis (Bai et al., 2021). Antibiotic treatment and microbiota transplantation experiments further demonstrated that remodelling of the gut microbiota is required for the bioactivity of oroxylin A. Oroxyloside (Oroxylin A 7-O-glucuronide) is one of the main metabolites of oroxylin A (Chen et al., 2000; Kim et al., 2012; Yao et al., 2014). *In vivo* experiments demonstrate the efficacy of oroxyloside in specifically attenuating pathological damage in colon (Wang et al., 2016). Oroxyloside decreases the secretion of IL-1β, IL-6, and TNF-α, whereas this reduction is reversed by the presence of GW9662, a specific inhibitor of PPARγ. PPARγ acts as an influential pleiotropic regulator of anti-inflammatory, antioxidant, and phagocyte-mediated clearance processes (Su et al., 1999; Desreumaux et al., 2001; Cevallos et al., 2021), and it plays an important role in the physiological function of the gastrointestinal tract (Sarangdhar et al., 2021). These studies showed that natural products can reduce the abundance of bacteria that disrupt the mucus layer and increase the level of TJs, reversing the disruption of the gut mucosal barrier in the early stages of CRC tumorigenesis. The PPARγ signalling pathway plays an important role in the effects of these natural products that depend on the gut microbiota to regulate the intestinal epithelial barrier.

It is of great significance to regulate the intestinal flora and improve the TME in order to prevent and treat tumour occurrence and development. Resveratrol, which is a ligand for the aryl hydrocarbon receptor (AhR),can shift the differentiation of T cells to promote Treg differentiation instead of Th17 differentiation through receptor-ligand interactions (Singh et al., 2007). The polarization of Th17 cells toward an inflammatory state triggers tumorigenesis in CRC (Gálvez, 2014). Interestingly, recent research has shown that Th17 cells are greatly influenced by the microbiome (IvanovAtarashi et al., 2009; Atarashi et al., 2011). Moreover, experiments on mice with IBD proved that resveratrol can decrease the abundance of *B. acidifaciens*, triggering a strong inflammatory cascade response, which as expected, includes the activation of IL-17-dependent pathways (Chung et al., 2018; Alrafas et al., 2019), and attenuating colorectal inflammation. However, another experiment presented different results. Ginsenosides Rb3 and Rd promote the growth of beneficial bacteria, such as *Bifidobacterium spp*, *Lactobacillus spp*, *Bacteroides acidifaciens*, and *Bacteroides xylanisolvens*, while decreasing the abundance of cancer cachexia-associated bacteria, such as *Dysgonomonas* spp. and *Helicobacter spp*. All these changes were correlated with the regulation of proinflammatory cytokine production (Huang et al., 2017). A study on the role of Gegen Qinlian decoction in enhancing the effect of PD-1 blockade in CRC also confirmed the protective effect of *Bacteroides acidifaciens* (Lv et al., 2019). Parthenolide (PTL), a sesquiterpene lactone originally extracted from the shoots of the plant Feverfew (*Tanacetum balsamita*), has been shown to exert potent anticancer and anti-inflammatory effects (Ren et al., 2019; Freund et al., 2020). PTL-treated mice exhibit increased SCFA production, and PTL administration selectively increases the frequency of colonic regulatory T (Treg) cells and decreases the proportion of colonic T helper type 17 (Th17) cells. Notably, PTL’s protective effect on colon inflammation disappeared when the gut microbiota is depleted using antibiotic cocktails (Liu et al., 2020a). Moreover, FMT confirmed this gut microbiota-dependent mechanism of action of PTL. As mentioned above, *F. nucleatum* potentiates intestinal tumorigenesis by modulating the tumour-immune microenvironment in mouse models (Kostic et al., 2013). Previous studies have reported that berberine (BBR) (Wu et al., 2012) exerts a preventative effect on colonic tumorigenesis. It has been proven BBR can reverse the *F. nucleatum*-induced imbalance of luminal microbiota and colon tumorigenesis in mice (Yu et al., 2015). These results suggest that natural products are potential therapeutic strategies for ameliorating the inflammatory-cancer transformation of CRC by modulating the gut microbiota, increasing the SCFA content, and regulating the Treg/Th17 balance. Targeting the role of microbes in the TME could improve our ability to prevent the development of cancer and activate the immune system to eliminate existing malignancies.

A number of studies have shown that natural products can inhibit CRC tumorigenesis through an integrated mechanism that involves multiple processes (Figure 1B). Natural products regulate the composition of the gut microbiota, improve immunity by increasing beneficial bacteria and reducing harmful bacteria, modulate immune cell function and reduce inflammatory responses. Natural products can reduce the abundance of known promoters of multiple processes, such as *Escherichia coli*, Fn, and *Bacteroides fragilis*, and mediate CRC transformation by targeting both the intestinal epithelial barrier and the TME. Interestingly, the classification of healthy and unhealthy flora requires caution. More research is needed to elucidate the molecular mechanisms by which “dual-identity” bacteria elicit different responses. The complex balance between the gut microbiota and host immunity not only affects tumorigenesis but also modulates antitumor effects of personalized cancer treatments.

### The effect of natural products on chemotherapeutic effects mediated by the gut microbiota

Currently, the main treatments for CRC are surgery, chemotherapy, radiotherapy, and targeted therapy. Among these options, chemotherapy is the main treatment for advanced or metastatic CRC, and chemotherapy is based on 5-fluorouracil (5-Fu) and platinum (Dekker et al., 2019). However, patients who benefit from 5-Fu-based therapy are prone to develop chemoresistance and are affected by haematopoietic and gastrointestinal toxicities (Blondy et al., 2020; Vodenkova et al., 2020). Hence, we need a new strategy to increase the efficacy of 5-Fu, overcome resistance and reduce nonspecific toxicity.

Evidence shows that the gut microbiota can regulate the effects of chemotherapeutic drugs in *in vivo* and *in vitro* models of CRC (Alexander et al., 2017; Roberti et al., 2020). Fn, which is an anaerobic parasitic bacterium, is increasingly related to CRC, and it has been demonstrated that Fn can promote chemoresistance to 5-Fu (Yu et al., 2017b; Zhang et al., 2019b). Moreover, metabolites of the gut microbiota also have the same effect. A study found that butyrate could promote the effect of oxaliplatin by modulating CD8^+^ T cell function in the TME by activating the IL-12 signalling pathway (He et al., 2021). On the other hand, many chemotherapeutic drugs, such as 5-Fu, can lead to intestinal damage and alter the diversity of the gut microbiota (Sougiannis et al., 2019; Zhang et al., 2020). Based on these conditions, natural products can enhance the beneficial effects of chemotherapy and reduce the adverse events caused by chemotherapy by modulating the gut microbiota.

**FIGURE 2:**
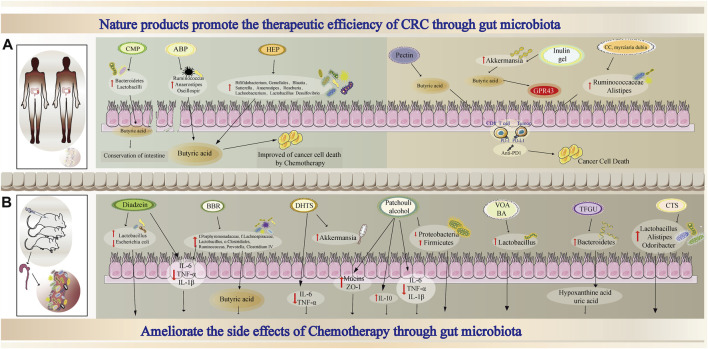
Proposed mechanism combined nature products and chemotherapy or immunotherapy through gut microbiota in oncotherapy **(A)** The combination of nature products and chemotherapy or immunotherapy enhances tumor therapeutic effect through gut microbiota **(B)** Nature products ameliorate the side effects of chemotherapy through gut microbiota.

Many studies have investigated whether natural products enhance chemosensitivity and modulate the gut microbiota to reduce the adverse events caused by chemotherapeutic drugs in tumour-bearing models. Albuca bracteate polysaccharides (ABPs), which have been reported to exert anti-inflammatory, antioxidant and antitumor effects, synergistically exerted antitumor effects with 5-Fu in CT-26 tumour-bearing mice. The combination treatment resulted in dramatically higher relative abundances of *Ruminococcus*, *Anaerostipes*, and *Oscillospira* (Yuan et al., 2021). In addition, the levels of butyric acid, which is a beneficial SCFA, were higher than those in the 5-Fu treatment group. The combination of another natural product, carboxymethyl pachyman (CMP), with 5-Fu reversed the intestinal shortening and ameliorated the colon injury induced by 5-Fu in CT-26 tumour-bearing mice. Furthermore, CMP can regulate the dysbiosis of the gut microbiota caused by 5-Fu by notably increasing the abundance of *Bacteroidetes*, *Lactobacilli*, and butyric acid-and acetic acid-producing bacteria (Wang et al., 2018b). Similarly, other natural products also exert the same effects (Wang et al., 2018b; Wang et al., 2020b). Thus, the combination of 5-Fu with natural products may maximize the antitumor effect and attenuate the intestinal changes caused by 5-Fu (Figure 2A).

In addition to tumour-bearing mouse models, studies also showed that natural products could attenuate adverse events in 5-Fu-treated models. BBR, which has been used to prevent colorectal adenoma (Chen et al., 2020a), exerts a protective effect in the intestine to ameliorate 5-Fu-induced intestinal mucositis by modifying the gut microbiota in tumour-free rats (Chen et al., 2020b). This study showed that BBR could significantly increase the levels of butyrate and glutamine in faeces. In addition, compared to 5-Fu-treated rats, rats treated with BBR plus 5-Fu show decreased diarrhoea scores, reduced inflammatory responses in the ileum and alleviated intestinal mucosal injury in faecal transplantation experiments, and these changes may be associated with increased butyrate levels among faecal metabolites (Chen et al., 2020b). Similarly, a study evaluated the effects of a volatile oil from *Amomum villosum* (VOA) and its main active compound bornyl acetate (BA) on intestinal mucositis induced by 5-Fu. Both VOA and BA relieve the effects of 5-Fu, enhance the intestinal mucosal barrier and increase the abundance of probiotics, such as *Lactobacillus* (Zhang et al., 2017b). Furthermore, many other natural products exert the same effects observed in the 5-Fu-induced model (Atiq et al., 2019; Wang et al., 2020c; Wu et al., 2020). In addition, not every natural product has the potential to improve chemotherapy-induced diarrhoea. A study showed that neither *Atracylodes macrocephala* essential oil (AMO) nor *Panax ginseng* total saponins (PGS) alone could obviously improve the abnormalities caused by 5-Fu, which include diarrhoea and pathological changes in the ileum and colons. However, the combination of these two natural products suppresses the production of inflammatory cytokines in the intestine, restore the ratios of *Firmicutes/Bacteroidetes* (F/B) and reduce the abundance of potential pathogens (Wang et al., 2019). This study indicated that the combination of natural products might be a promising strategy for treating chemotherapy-induced damage.

In addition to 5-Fu, irinotecan (CPT-11) can cause diarrhoea in clinical practice because symbiotic bacterial β-glucuronidases reactivate the drug in the gut (Wallace et al., 2010). However, natural products also improved the diarrhoea caused by CPT-11 in a mouse model. Total flavonoids of *Glycyrrhiza uralensis* (TFGU) exert a protective effect on CPT-11-induced colitis by inhibiting proinflammatory responses and reverse faecal metabolic disorders, including the metabolism of hypoxanthine, uric acid, and purine (Yue et al., 2021). Moreover, crypotanshinone (CTS) relieves 5-Fu/CPT-11-induced colitis in a model of colitis-associated colon cancer (CAC) by regulating the gut microbiota, and this effect is correlated with effects on lipid metabolism (Wang et al., 2021b). Thus, natural products may modulate different chemotherapeutic drug-induced intestinal pathological changes *via* the gut microbiota and its metabolites.

Taken together, these data suggest that natural products can exert synergistic effects with chemotherapy drugs and alleviate adverse effects by modulating the gut microbiota and its metabolites (Figure 2B). Based on these characteristics and advantages, nature products can be a promising part of combined therapies.

### The effect of natural products on immunotherapy mediated by the gut microbiota

In addition to the treatments we mentioned, the emergence of immunotherapy has provided a transformative new method for the comprehensive treatment of cancer. Tumour immunotherapies mainly include ICIs, cellular immunotherapy and immune vaccines; in particular, ICIs are widely used in clinical practice. Based on studies, such as KEYNOTE-177 and CheckMate-142, immunotherapy has been recommended by the 2021 NCCN guidelines for the treatment of advanced or metastatic CRC with MSI-H/dMMR (Overman et al., 2017; André et al., 2020; Andre et al., 2021). However, the incidence of dMMR CRC is approximately 5%, and the response rate ranges between 30 and 50% (Gurjao et al., 2019; Sahin et al., 2019; Vasaikar et al., 2019); thus, researchers have focused on the combination of VEGF inhibitors, chemotherapy, and many other specific inhibitors (Guangdong Provincial People’s Hospital, 2022; Bristol-Myers, 2022; Tianjin Medical University Cancer Institute and Hospital, 2021). Moreover, natural products have shown promises as immunomodulatory agents. Studies have shown that compared to ICIs alone, the combination of ICIs and natural products can exert synergistic effects on CRC by promoting antigen presentation, enhancing CD8^+^ T cell cytotoxic activity, increasing T cell infiltration and so on (Liu et al., 2020b; Liu et al., 2020c; Lee et al., 2021; Xu et al., 2021). This may be a new strategy to transform “cold” tumours into “hot” tumours.

Currently, the gut microbiota has attracted the attention of researchers in terms of its role in antitumor immunotherapy (Cremonesi et al., 2018; Zitvogel et al., 2018; Bouferraa et al., 2021; Matson et al., 2021; Xing et al., 2021). Researchers have adopted multiple mouse models or faecal microbiota transplantation (FMT) models to reveal the relationship between the gut microbiota and ICIs (Sivan et al., 2015; Vétizou et al., 2015; Yi et al., 2018; Elkrief et al., 2019; Zhang et al., 2022). These studies suggest that both the commensal gut microbiota and that of healthy people play an important role in the immune microenvironment. This was also true in a clinical model. V. Gopalakrishnan et al. (Gopalakrishnan et al., 2018) reported that melanoma patients who responded to anti-PD-1 blockade had high abundance of *Faecalibacterium* species, and this population had longer PFS and higher levels of effector CD4^+^ and CD8^+^ T cells in systemic circulation. Similarly, the abundance of *B. fragilis* was related to the efficacy of CTLA-4 blockade in melanoma patients and an FMT model (Vétizou et al., 2015). Additionally, other studies described the characteristics of the gut microbiota of different patients who responded to ICIs to predict the outcomes of immunotherapy in patients with NSCLC, hepatocellular carcinoma and gastrointestinal cancer (Jin et al., 2019; Zheng et al., 2019; Peng et al., 2020b). Metabolites of the gut microbiota have also become biomarkers to predict beneficial outcomes of ICI treatment (Mager et al., 2020). Since the gut microbiota affects ICI efficacy and natural products also influence the gut microbiota and immunotherapeutic efficacy as described above, we next asked whether natural products can exert a synergistic effect with immunotherapy by affecting the gut microbiota.

First, natural products can enhance sensitivity to immunotherapy by altering the diversity of the gut microbiota and its metabolites. Inulin combined with anti-PD-1 can significantly increase the relative abundances of *Akkermansia*, *Lactobacillus* and *Roseburia*, which have been reported to be associated with ICI-responsiveness in patients. When the form of the drug administered was changed to an inulin gel, inulin gel plus anti-PD-1 therapy increased the relative abundance of *Akkermansia* and resulted in an increasing trend in the abundance of *Roseburia*. Combined treatment can increase the abundance of the beneficial commensal microbiota and SCFA metabolites to increase Tcf1^+^PD-1^+^CD8^+^ T cell numbers and improve CRC tumour burden (Han et al., 2021). Similarly, dietary consumption of *Lactobacillus*-derived exopolysaccharide increases CCR6+CD8^+^ T cell numbers in Peyer’s patches and enhances ICI therapeutic effects to improve the TME (Kawanabe-Matsuda et al., 2022). Although there are few studies on the effect of the gut microbiota and its metabolites on the efficacy of ICIs in patients with CRC, there are many studies about this topic in other cancers. For example, in patients with melanoma or NSCLC, ginseng polysaccharides (GPs) combined with anti-PD-1 therapy alters the gut microbiota and increases the abundance of SCAFs, especially valeric acid, but not acetic acid. In addition, GPs affect the ratio of kynurenine/tryptophan through gut microbes to increase the response to anti-PD-1, which suppresses Tregs and activates effector T cells (Huang et al., 2021).

Second, in addition to improving sensitivity to immunotherapy, natural products can reverse CRC resistance to ICIs by modulating the balance of the gut microbiota. Polyphenol-rich berry camu-camu (CC, *Myrciaria dubia*), which exerts no antitumour effect when administered orally, exerts a synergistic effect when combined with αPD-1 mAb. In addition, oral administration of castalagin, which is an active compound of CC, can enrich bacterial species associated with efficient immunotherapeutic responses (*Ruminococcaceae* and *Alistipes*) and enhance the CD8^+^/FoxP3^+^CD4^+^ ratio in the TME; additionally, it can act as a prebiotic to circumvent anti-PD-1 resistance (Messaoudene et al., 2022). Similarly, pectin reverses the effect of anti-PD-1 in humanized tumour-bearing mice transplanted with the gut microbiota from CRC patients and promotes T cell infiltration and activation (Zhang et al., 2021). Moreover, the results demonstrated that in the individuals administered the combination treatment, unique bacterial modules composed of butyrate-producing bacteria exhibit a better response to immunotherapy. These studies reveal that natural products can reverse CRC resistance to ICIs by modulating the balance of the gut microbiota and provide a new potential approach for overcoming immune resistance.

Finally, ICIs are associated with clinical benefits across cancer types but may be accompanied by adverse events to some extent. A study demonstrated that the abundance of *Bacteroides intestinalis* is correlated with IL-1β production and toxicity caused by the combination of CTLA-4 and PD-1 blockade in patients with melanoma (Andrews et al., 2021). However, more preclinical experiments and clinical practice are still needed to ameliorate the toxicity associated with ICIs; in particular, larger sample sizes are required to explore the alteration of the gut microbiota under these conditions.

## Conclusion and perspectives

The development of CRC is a complex pathophysiological process, and the gut microbiota plays an essential role in both inflammation-induced tumour development and tumour treatments. The gut microbiota, which can be used as a biomarker or a prognostic factor, has also become a new target for identifying responses to disease development and treatments. Similarly, natural products, which have the advantages of being easily available, being widely used, and having multiple targets, have also become a promising method for preventing the development of disease, reducing inflammation, modulating immunity and reversing resistance.

The current research about the prevention of tumorigenesis by natural products and the intestinal flora has highlighted the factors involved in the activation of signalling pathways and improvements in functions that were used to initially elucidate the underlying mechanisms and develop new treatment strategies. Antibiotic depletion and FMT were used to validate causal relationships between natural products and the gut microbiota; the transplantation of the faecal microbiota from donors with diseases who had received treatments protect mice against colitis (Liu et al., 2020a) or colon carcinogenesis (Parker et al., 2021). This indicates the potential role of natural products in preventing tumorigenesis by modifying the intestinal microflora. However, further FMT experiments involving healthy donors and healthy donors treated with natural products (Sui et al., 2020) are needed to explore the complex relationship between natural products and the microbiota. Recent studies have revealed the existence of interactions between the host and intestinal flora (Yang et al., 2018). Currently, representative sets of multiomics studies (Lloyd-Price et al., 2019; Mars et al., 2020) are available for the in-depth analysis of the gut microbiota to understand the molecular mechanisms by which it affects tumorigenesis and tumour progression. These efficient experimental strategies will contribute to the development of effective treatments, which will ultimately prevent tumorigenesis.

The present study provides insights into CRC therapy. There is a delicate balance between epithelial, microbial, and immune cell interactions, and disruption or deviation from this balance can lead to risks of tumour promoting. The use of natural products in treatments allows multiple molecules and processes to be targeted and is a valuable clinical approach for preventing tumorigenesis. These targets include remodelling the normal host/microbial symbiosis system, maintaining the intestinal epithelial mucosal barrier, inhibiting tumour-promoting T regulatory cells, Th17 cells and inflammatory cells, and improving the TME. Natural products can regulate the intestinal flora, improve the efficacy of chemotherapy or immunotherapy, and reduce the adverse effects of some chemotherapy drugs. With the development of additional targeted drugs and immunotherapeutic approaches, researchers are exploring drug combinations to promote optimal antitumor effects. This provides prospects for the potential clinical application of natural products in preventing inflammation from transforming into CRC and in comprehensively treating CRC.

## Glossary

CRC: Colorectal cancer
IBD: Inflammatory bowel disease
UC: Ulcerative colitis
CD: Crohn’s disease
NF-κB: Nuclear factor-kappa B
IL-6: Interleukin 6
TNF-α: Tumor necrosis factor alpha
PUFAs: Polyunsaturated fatty acids
ICIs: Immune checkpoint inhibitors
AIEC: Adherent-invasive *E. coli*
AOM: Azoxymethane
DSS: Dextran sulphate sodium
GF: Germ-free
LPS: Lipopolysaccharide
LBP: Lipopolysaccharide-binding protein
MD-2: Myeloid differentiation factor 2
TLR4: Toll-like receptor 4
MyD88: Myeloid differentiation primary response 88
IL-1β: Interleukin 1beta
Cdk5: Cyclin-dependent kinase 5
CRAMP: Cathelin-related antimicrobial peptide
TJs: Tight junctions
APC: Adenomatous polyposis coli
DCs: Dendritic cells
MDSCs: Myeloid-derived suppressor cells
ETBF: Enterotoxic *Bacteroides fragilis*
CECs: Colonic epithelial cells
IL‐17: Interleukin 17
STAT3: Signal transducer and activator of transcription 3
MMP‐9: Matrix metalloproteinase 9
VEGF: Vascular endothelial growth factor
Lp: *Lactobacillus* plantarum
NK cells: Nature killer cells
IL‐22: Interleukin 22
GLP: Ganoderma lucidum
AG: astragalin
ASD: Akebia saponin D
PTL: Parthenolide
BBR: berberine
CAC: Colitis-associated cancer
COX-2: Cyclooxygenase-2
MCP-1: Monocyte chemoattractant protein-1
IFN-γ: Interferon-gamma
PPARγ: Peroxisome proliferator-activated receptor gamma
AhR: Aryl hydrocarbon receptor
SCFAs: Short-chain fatty acids
5-Fu: 5-fluorouracil
TME: Tumor microenvironment
IL-12: Interleukin 12
CPT-11: Irinotecanl
NCCN: National Comprehensive Cancer Network
MSI-H: Microsatellite instability High
dMMR: Defective DNA mismatch repair
FMT: Fecal microbiota transplantation
CTLA-4: Cytotoxic T-lymphocyte associated protein 4
PD-1: Programmed cell death 1
NSCLC
Non-small-cell lung cancers
ABPs: Albuca bracteate polysaccharides
CMP: Carboxymethyl pachyman
VOA: a volatile oil from Amomum villosum
BA: Bornyl acetate
AMO: Atracylodes macrocephala essential oil
PGS: Panax ginseng total saponins`
TFGU: Glycyrrhiza uralensis
CTS: Crypotanshinone
GPs: Ginseng polysaccharides
CC: Myrciaria dubia: Polyphenol-rich berry camu-camu.
